# Protozoan Parasite *Toxoplasma gondii* Manipulates Mate Choice in Rats by Enhancing Attractiveness of Males

**DOI:** 10.1371/journal.pone.0027229

**Published:** 2011-11-02

**Authors:** Shantala Arundathi Hari Dass, Anand Vasudevan, Deborah Dutta, Linda Jing Ting Soh, Robert Morris Sapolsky, Ajai Vyas

**Affiliations:** 1 School of Biological Sciences, Nanyang Technological University, Singapore, Republic of Singapore; 2 Department of Biology, Stanford University, Stanford, California, United States of America; Institut national de la santé et de la recherche médicale - Institut Cochin, France

## Abstract

Females in various species typically avoid males infected with parasites, while parasite-free males advertise their status through conspicuous phenotypic traits. This process selects for heritable resistance and reduces direct exposure of the female to parasites. Coevolving parasites are likely to attempt to circumvent this obstacle. In this paper, we demonstrate a case of parasitic manipulation of host mate choice. We report that *Toxoplasma gondii*, a sexually transmitted infection of brown rats, enhances sexual attractiveness of infected males. Thus under some evolutionary niches, parasites can indeed manipulate host sexual signaling to their own advantage.

## Introduction

Host and parasites coevolve by placing constant demand on each other for adaptations and counter-adaptations. Selection pressures acting on the host can be very severe, in fact severe enough to drive host towards bi-parental reproduction even when this is very costly to maintain non-childbearing males [1]. Hence, it is not surprising that females typically avoid and in fact show aversive response to parasitized males [2–12]. Aversion of females to parasitized males is likely driven by the evolutionary need for females to choose for heritable parasite resistance [13]; and/or avoid direct transmission of contagious diseases during mating [14]. Such female aversion is detrimental for parasites, particularly if they are transmitted by sexual intercourse. We posit that in this situation, parasites have an evolutionary pressure to manipulate host males in a way that overcomes the traditional female aversion.

We tested this hypothesis using a common protozoan parasite of the brown rats, *Toxoplasma gondii* [15]. Recently, *Toxoplasma gondii* has been reported to be sexually transmitted in sheep and dog [16,17]. We first tested if it is a sexually transmitted in rats; we then investigated if chronic *Toxoplasma gondii* infection manipulated female mate choice.

## Results

### *Toxoplasma gondii* is a sexually transmitted infection in brown rats

**Figure 1:**
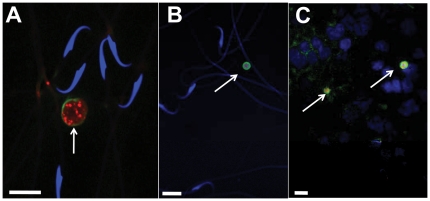
*Toxoplasma gondii* is transmitted through intercourse. *Toxoplasma gondii* cysts were observed in epididymis of infected males (panel A), in vaginal lavage of naïve females mated with infected males (panel B) and in brain of pups derived from these mating (panel C). Scale bar = 10 µm. Parasites are stained red (anti-GFP antibody couples with fluorogenic detection using Cy3), cyst wall green (dolichos biflorus agglutinin coupled with fluorescein) and sperm nuclei in blue (DAPI).

Parasitic cysts containing bradyzoites could be visualized eight weeks post-infection in epididymis, a tubular structure that collects and stores sperm (estimated burden  = 520 cysts per animal, 3–7 µm in diameter, n = 5 animals; Figure 1A). These cysts were viable; feeding them resulted in sero-conversion of uninfected females (4/4 attempts; one infected epididymis/female). Infected males were mated with uninfected females. *Toxoplasma gondii* cysts could also be observed from vaginal lavage of females 12 hours after mating (6 out of 7 mating; Figure 1B), indicating that *Toxoplasma gondii* was successfully ejaculated. Mating with an infected male resulted in transmission of infection to females (4/4 mating). This was demonstrated by successful PCR amplification from female brain of a gene unique to the parasite. Using previously published primer sets, we successfully amplified B1 gene of *Toxoplasma gondii* from genomic DNA prepared from crude lysate of female brains four weeks after mating (see [18] for methods). Identity of PCR product was confirmed using DNA sequencing. Infection was also confirmed by serological tests showing presence of anti-parasite IgG antibodies in mated females (serum dilution 1∶1000; 6/7 mating). Serum obtained from mated females contained primary antibodies against laboratory-cultured *Toxoplasma* gondii tachyzoites (visualized using anti-rat IgG coupled with Cy3). Mated females were allowed to give birth, and pups were tested for presence of *Toxoplasma gondii*. Parasite cysts were detected in 43 out of 69 pups that we tested (Figure 1C). Hence *Toxoplasma gondii* is a sexually and vertically transmitted infection in rats.

### Reproductive parameters of control and infected animals were comparable

Despite invading reproductive organs, *Toxoplasma gondii* did not adversely affect sexual behavior or fecundity/fertility of infected males. We tested the attraction of sexually naïve control and infected males to females. Attraction was quantified by comparing time spent by a male in two opposing arms of an arena, each arm containing either an inaccessible estrus female or an unfamiliar male. Both control and infected animals exhibited comparable attraction to females (n = 11 males in each group; exact Mann-Whitney test: Z = −0.23, *p*>0.8; % time spent in bisect containing receptive female  = 58±4.8% for control and 59±3.7% for infected animals). Control and infected animals exhibited comparable number of mounts and intromissions during paced mating with uninfected females (n = 6 control and 7 infected males; exact Mann-Whitney test: Z<−0.43, *p*>0.7; Table 1). Likewise, infection did not affect the number of pups, weight of newborns or sex ratio of progeny resulting from mating (Table 2).

**Table 1:** Despite invading reproductive tissue, *Toxoplasma gondii* did not affect mating performance of infected animals.

	Group	N	Mean	Std. Deviation	Std. Error Mean	*p*-value
Mounts	Control	6	129.67	65.347	26.678	0.731
	Infected	7	119.86	40.429	15.281	
Intromissions	Control	6	110.50	51.130	20.874	0.945
	Infected	7	116.43	39.782	15.036	

Control and infected males were mated with estrus female in a paced mating set-up, whereby female could pace the sexual interaction. Both control and infected animals performed comparably.

**Table 2:** Despite invading reproductive tissue, *Toxoplasma gondii* did not affect number and sex ratio of progeny.

	Group	N	Mean	Std. Deviation	Std. Error Mean	*p*-value
Body weight of newborn pups	Control	83	6.266	0.4374	0.0480	0.04
	Infected	76	6.414	0.5230	0.0600	
# of Pups per mating	Control	6	14.17	0.983	0.401	0.47
	Infected	6	13.00	2.191	0.894	
Sex Ratio (% Male Pups)	Control	6	39.433	23.3232	9.5216	0.69
	Infected	6	48.883	9.7183	3.9675	

Infected animals sired comparable number of live progenies with similar sex ratio. Pups born to infected fathers weighed marginally more, an effect statistically significant but of very small effect size.

### Uninfected females preferred infected males

**Figure 2:**
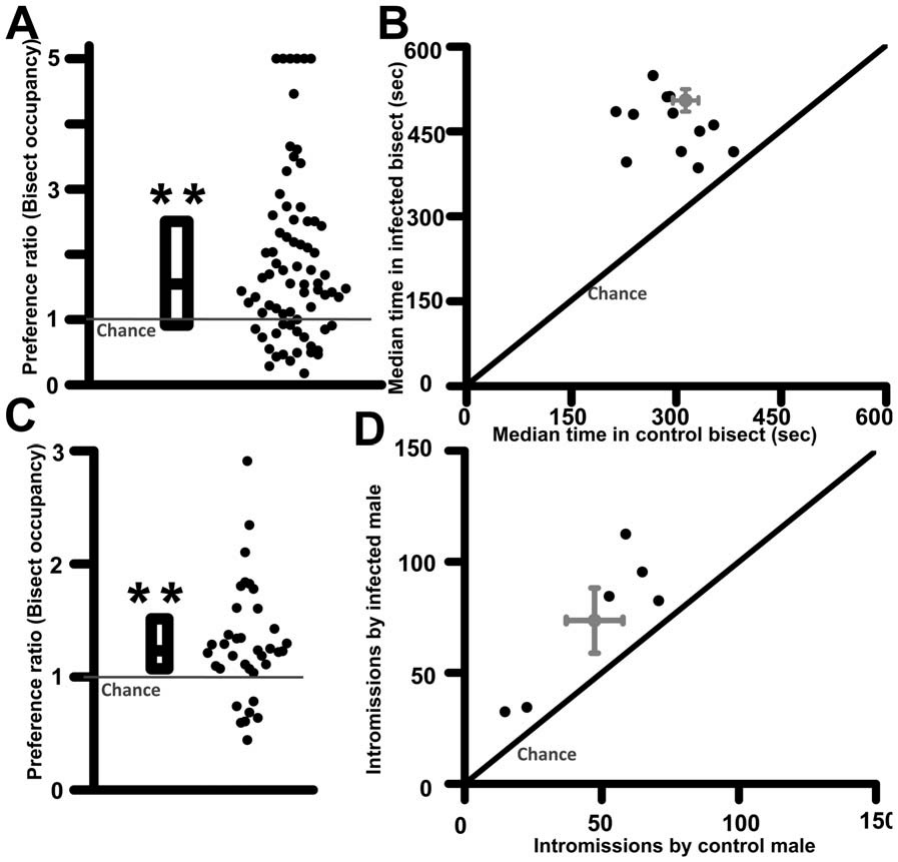
Uninfected females preferred infected males. Preference was quantified by comparing time spent by an estrus female in two opposing arms of an arena, each arm urine marked by either controls or males infected six weeks earlier (panel A; trial duration  = 1200 s). Ordinate depicts time spent in infected bisect divided by control bisect (ratio >5 assigned arbitrary value of 5). Each dot represents raw data from one female. Box plots depict median, 25^th^ percentile and 75^th^ percentile. Preference of females for males in each of the 12 unique pair was calculated by taking median of all females tested for that particular male (panel B; ordinate and abscissa depicts time spent in infected and control bisect, respectively). Mean and SEM of data used in scatter-plot are depicted in grey color. These results were confirmed using a different rat strain in a geographically distinct location (panel C; Long-Evans rats in SU, USA; in place of Wistar rats at NTU, Singapore). **, *p*<0.001; Chi^2^ test. In a competitive paced mating, females chose to participate in more mounts and intromissions with infected males (panel D; ordinate and abscissa depicts number of intromissions by infected and control males, respectively). *, *p*<0.01; one-sample t-test against chance, i.e. 1. Intromission data exhibited one outlier that has been removed from the graph. Mean and SEM are depicted in grey color.

Next, we tested if uninfected females avoided parasitized males, as would be expected from the extensive literature on this subject [2–12]. Preference of an estrus female for control or infected male was determined during a two-choice preference task (12 pairs of males, 5–7 females per pair; total 72 trials). Contrary to expectation, sexually naïve females spent more, rather than less time in the infected bisect (Wilcoxon signed ranks test: Z = −4.547, *p*<0.00001; 506±30 s in infected bisect versus 308±17 s in control; n = 72 trials). A preference score of females for infected males was computed for each trial by dividing time spent in infected bisect by that in control. During 74% of trials, females spent more time in the infected bisect (Figure 2A; Chi^2^ test: Chi^2^ = 16.1; *p*<0.0001; infected>control  = 53 trials, control >infected  = 19 trials; median preference score  = 1.54).

Time spent in infected and control bisects for each unique male pair was calculated by taking median of all females tested for each male pair. In all male pairs tested, median occupancy in infected bisect was greater than median occupancy in control area (Figure 2B; exact Wilcoxon signed ranks test: Z = −3.059; *p*<0.0001).

The generality of this finding was confirmed using a different strain of rats in a geographically different laboratory (Long-Evans rats in Stanford University; Figure 2C; n = 36 females; Chi^2^ test: Chi^2^ = 13.4; *p*<0.001; infected>control  = 29 females, control >infected  = 7 females; median preference score  = 1.30). In this case, male bedding was used instead of urine marks and males were absent from the arena during testing. Since males were not physically present during this experiment, this precludes a role for male vocalization in our observations.

### Infected males gained greater reproductive opportunities

In a competitive paced mating set-up whereby female chose to mate between one control and one infected male, females allowed greater number of reproductive opportunities to infected males (Figure 2D). During two-hour trial, infected Wistar males secured more intromissions compared to controls (n = 6 pairs; exact Wilcoxon signed ranks test: Z = −2.21, *p*<0.05; one-sample t-test against chance i.e. 1: t = 4.36, *p*<0.01). Hence, *Toxoplasma gondii* infection altered mate choice of females in a completely unexpected direction, whereby infected male gained in terms of reproductive fitness.

## Discussion

### Further support for manipulation hypothesis

The core of parasitism is the ability of an organism to exploit its host. According to the behavioral manipulation hypothesis, a parasite may be able to alter the behavior of its host for its own selective benefit [19–22]. *Toxoplasma gondii* has been previously described as a classical case of parasitic behavioral manipulation [23–26]. It blocks the aversion of rats for cat urine, instead producing an attraction. This behavioral change is likely to increase the likelihood of a cat predating a rat. This is thought to reflect an adaptive behavioral manipulation by the parasite because it reproduces sexually only in the gut of the cat [23,24,27]. In this report, we show that *Toxoplasma gondii* is transmitted through sexual intercourse in brown rats, and that the parasite can manipulate mate choice of uninfected females. This behavioral change plausibly enhances transmission of the parasite from males to females and their progeny. Data presented here suggest a novel class of behavioral manipulation by *Toxoplasma gondii*. This runs counter to well-established observations in rodents [4,6] and in other animals [2,3,5,7–11] that females detect and avoid males infected with an array of viruses, bacteria, protozoa and nematodes. For example, female mice avoid males infected with *Eimmeria vermiformis*, a close relative of *Toxoplasma gondii*, and have an aversive physiological reaction to the smell of such males [6]. As a note of caution, our evidence for sexual/vertical transmission is restricted to our examination of seven matings between infected males and uninfected females, four females post-mating and sixty-nine progenies, under laboratory conditions. This is supported by evidence of sexual transmission in sheep and dogs [16,17]. This observation is in need of verification from other research groups and in larger-scale field experiments. This has important implications because of our assumption that sexual transmission is frequent enough to produce selection pressure for a parasitic manipulation.

The notion of extended phenotype refers to the situations where genotype of an organism manifests its phenotype outside the physical confines of its body [28]. Parasitic behavioral manipulation is an elegant demonstrations of extended phenotypes. In this case, genotype of the parasite induces a phenotype in terms of host behavior. The ability of the *Toxoplasma gondii* to not only advantageously alter the behavior and physiology of its host, but to also secondarily alter the behavior of uninfected females presents a striking example of the "extended phenotype” [28].

We suggest that enhanced attractiveness of infected rats is a parasitic manipulation meant to increase chances of parasite transmission. This suggestion should be carefully tested against the ease of erroneously invoking an adaptationist explanation (see [20] for scholarly discussion of the issue). A behavioral manipulation is “too well fitted” to its purpose to arrive by chance. In other words, there needs to be a conformity between the observed change and “a priori design specification” that a engineer might use [20]. For example, both increase and decrease in locomotion of prey can be argued to lead to increased predation; it is not purposive [20]. In this regard, an infection that imparts more attractiveness to male is an “a priori design specification”; an obvious solution to get more parasites travel with semen. Thus, we posit that the behavioral change reported here constitutes a behavioral manipulation rather than a generalized effect of infection. Nonetheless, this view makes an important assumption that sexual transmission plays a sizeable part in life history of the parasite, an assumption that needs to be tested further in large-scale field experiments.

### Exploitation of host sexual selection

Various theories have been proposed to explain the functional significance of mate choice (reviewed in [29–31]); based on direct benefits to the female [32], heritable parasite resistance [13], prevention of associative transmission of infections [14] or runaway selection processes [33]. Regardless of selection pressure that maintains mate choice, it remains beneficial for a sexually transmitted parasite to manipulate it. This possibility has not been studied extensively. On related note, male chironomid midges (*Paratrichocladius rufiventris*) have better success at forming mating pair if infested by a mite (*Unionicola ypsilophora*) [34]. Mating in midges generally involves females forming a swarm and male flying through it trying to capture a female. It is not clear if female mate choice or inter-sexual signaling plays any meaningful role in midges. Thus, possibility of parasitic effects on mate choice has remained unaddressed in this system.

Apart from parasitic manipulation of mate choice, there are two kinds of situations where host sexual signaling and parasites could interact. In one type of examples, parasites invade reproductive tissue of host producing ‘parasitic castration’ [35], aiming to block host from spending valuable metabolic currency on its own reproduction. In a second type of examples, parasites act as ‘illegitimate receivers’ of sexual signals in order to locate potential hosts [36]. Both of these situations are distinct from behavioral manipulation of mate choice.

Female mate choice is an important mediator of reproductive success of male rats. Several semi-naturalistic and laboratory studies have demonstrated that female rats strongly pace the sexual interaction through punctuated display of solicitation behavior (reviewed in [37], also see [38–40]). Indeed neuroendocrinological changes essential for initiating pregnancy depend on intermittent nature of coital stimulation in females. These observations suggest that solicitation controlled by females is central to rat sexual behavior. Thus, it is a plausible speculation that a parasitic strategy based on female mate choice will be selected.

Role of *Toxoplasma gondii* infection in rat mating success has been investigated before [41], using a semi-naturalistic setting. It was reported that infection did not alter mating success as defined by number of ejaculations and mounting. Reproductive success of males in this arrangement is a product of both male-male competition and mate choice. One possibility is that females in this arena had low opportunity to pace the interaction, for example by hiding in home-boxes that had an opening small enough to allow only females and not males. As indicated earlier, several studies have established that intermittent solicitation by females is regular feature of reproductive ritual in rats; and it facilitates successful pregnancy. Another possibility is that male-male competition heavily contributed to the mating success in semi-naturalistic setting, overriding influence of female mate choice. This possibility will require further experimentation in form of careful dissociation of both intra-sexual and inter-sexual behaviors. Pending that, it is difficult to ascertain if parasitic manipulation of mate choice will result in significant gain of reproductive fitness of infected males.

### Mechanism

What are the proximate mechanisms? It can be safely assumed that pheromonal communication rather than acoustic signaling is involved. This is because presence of soiled bedding itself is sufficient to show difference between infected and control males. Major urinary proteins play important part in sexual signaling of house mouse [42,43]. It is possible that change in similar proteins in rats is involved in manipulation of mate choice. This possibility is supported by the fact that rat urine contains large amount of major urinary proteins. On the other hand, mouse and rat major urinary proteins have evolved separately after divergence from a common ancestor [44]. Presently, there is no unequivocal evidence that rat major urinary proteins do participate in sexual signaling. Further experiments are needed to delineate pheromonal mechanisms involved in this behavioral change.

### Cost of the parasitism

There are varieties of documented cases where parasites manipulate host behavior in order to gain selective advantage [19–21]. Such behavioral manipulations usually have detrimental consequences for the host. But the atypical effects of *Toxoplasma gondii* on mate choice raise an intriguing speculation. Reproductive success in infected males is likely to be elevated, by virtue of their increased attractiveness. Potentially, this elevation could more than offset the decreased fitness of infected males due to their increased likelihood of being predated by cats. While difficult to test in studies of natural populations, these data raise the possibility that parasitic behavioral manipulation will at least blunt the cost of parasitism for infected males. It must be noted that precise determination of reproductive fitness is a difficult task, in view of insufficient quantitative knowledge about rodent reproduction and predation. Thus possibility that infection raises reproductive success of males itself is a speculation at this moment, albeit an interesting and plausible one (also see [41] for a contrary view).

Parasitic manipulation of sexual signaling is in contrast to the idea that sexual selection results in discrimination against parasitized males. Under some evolutionary niches, parasites can indeed manipulate host sexual signaling to their own advantage.

## Materials and Methods

### Animals

Wistar rats (48 days old, housed two/cage) were obtained from vivarium of National University of Singapore. The Nanyang Technological University institutional animal care and use committee reviewed and approved all procedures (ARF SBS/NIE- A0106AZ). Long-Evans rats (49 days old, three/cage) were obtained from Charles River laboratories (Willmington, MA). The Stanford University administrative panel for laboratory animal care approved procedures pertaining to Long-Evans rats (**APLAC#11603)**. Animals from this source tested serologically negative for *Toxoplasma gondii*.

### Parasites and Treatments

We used a Prugniaud strain genetically modified to constitutively express green florescent protein under GRA2 promoter. Parasites were maintained as tachyzoites by passage in human foreskin fibroblast monolayers [24,25]. Infected fibroblasts were syringe-lysed by using a 27-gauge needle to release tachyzoites. Animals were either infected with tachyzoites (5×10^6^, i.p.) or mock-infected with sterile phosphate buffered saline. It is noteworthy here that oral ingestion of oocysts or tissue cysts, and not intraperitoneal entry of tachyzoites, constitutes a more naturalistic route of infection. Yet, we have chosen to use tachyzoites because this produces a more consistent cyst burden in our experience with minimal risk of occupational exposure to laboratory personnel.

All behavioral experiments were conducted between 6 to 8 weeks post-infection, a period know to harbor chronic infection. Infected and control animals gained comparable weight during the experiment.

### Visualization of *Toxoplasma gondii* cysts

Smears from epididymis, post-mating vaginal lavage and brain tissue of fetus were examined for the presence of *Toxoplasma gondii* cysts. Taking advantage of endogenous production of GFP by the transgenic parasite, cysts were observed using anti-GFP antibody (Millipore Cat # AB3080, 1∶200 antibody dilution). Interaction between GFP and primary antibody was visualized using tyramide signal amplification coupled with Cy3 dye (red color; Perkin Elmer Cat # NEL744001KT**).** Cyst wall was stained using dolichos biflorus agglutinin (20 µg/ml) coupled with fluorescein (Vector Labs Cat # FL1031, green color). Sperm nuclei were visualized using DAPI (blue color). Stained smears were imaged using a confocal microscope (LSM 710 META, Zeiss).

### Polymerase chain reaction and Serological confirmation of infection status

We utilized polymerase chain reaction to selectively amplify a 35-fold repetitive sequence of the gene B1, a gene that is selectively found in *Toxoplasma gondii*. Primer sets and PCR methods were adopted from previously published protocols (5′-GGAACTGCATCCGTTCATGAG-3′ and 5′-TCTTTAAAGCTTCGTGGT C-3′) [18].

Infection was also confirmed by serological detection of anti-Toxoplasma antibodies. Toxoplasma was cultured inside human foreskin fibroblasts in 24-well plates. 24 hours post infection; the wells were aspirated, washed with PBS and fixed with 4% PFA. They were incubated with 1 ml of the serum (1∶1000) overnight at 4°C.If the animal had been infected with Toxoplasma the serum would contain anti Toxoplasma antibodies which would bind to Toxoplasma in culture. The bound antibodies were visualized by treating with anti Rat IgG-Cy3 (1∶200, Millipore) (red color).

### Mate choice assay

Naturally cycling females were used in all assays.

Wistar: Female mate choice was quantified by comparing time spent by estrus females in two opposing arms of an arena (76×9 cm each; 15 cm high) during a 20 minute trial. Opposing arms were scent marked by control and infected males. Males spent 60 minutes before the trial urine marking their respective bisects. Male were restricted to one end of bisect during actual trial, a perforated plastic sheet separating males and test female. Occupancy of female in each of the arm was quantified.

Long-Evans: Male stimulus consisted of 100 g of 24 h soiled bedding, pooled from 4 cages each housing three males; placed in opposing corners of a plastic box; 92.4×35.3 cm, 40.1 cm high. Occupancy of estrus female in bisects containing either control or infected odor was quantified.

### Paced mating

Non-competitive paced mating: Experiments were conducted in a glass arena with two chambers connected through a 5 cm semi-circular gate. One chamber housed either a control or an infected male (30×45 cm). At the start of trial, estrus female was placed in the arena and observed for duration of 2 hours. Because of difference in body-size, only female could cross the gate connecting two chambers; thus allowing female to pace sexual interactions by withdrawing in chamber not occupied by the male. Intromissions were defined as a reproductive sequence starting with mount and ending in self-genital licking by males.

Competitive paced mating: Arena similar to the one described above was used, but with two terminal chambers holding a control and an infected animals during same trial. Female could thus choose to have paced sexual encounter with either male or to withdraw in middle compartment where neither male was present.

### Statistical analysis

Mann-Whitney test was performed to determine statistical significance when comparing between experimental groups. Wilcoxon signed-rank test were used when comparing paired differences in within-subject parameters. Wherever appropriate, chi-square test was used to test deviation from ratios predicted by random occurrence. In all statistical tests, exact statistics was used to eliminate asymptotic and approximate statistical assumptions.
